# Natural biocide disrupts nestmate recognition in honeybees

**DOI:** 10.1038/s41598-019-38963-3

**Published:** 2019-02-28

**Authors:** Federico Cappa, Iacopo Petrocelli, Francesca Romana Dani, Leonardo Dapporto, Michele Giovannini, Jeferson Silva-Castellari, Stefano Turillazzi, Rita Cervo

**Affiliations:** Università degli studi di Firenze, Dipartimento di Biologia, Via Madonna del Piano 6, 50019 Sesto Fiorentino, Firenze Italy

## Abstract

Honeybee colonies are under the threat of many stressors, biotic and abiotic factors that strongly affect their survival. Recently, great attention has been directed at chemical pesticides, including their effects at sub-lethal doses on bee behaviour and colony success; whereas the potential side effects of natural biocides largely used in agriculture, such as entomopathogenic fungi, have received only marginal attention. Here, we report the impact of the fungus *Beauveria bassiana* on honeybee nestmate recognition ability, a crucial feature at the basis of colony integrity. We performed both behavioural assays by recording bee guards’ response towards foragers (nestmate or non-nestmate) either exposed to *B. bassiana* or unexposed presented at the hive entrance, and GC-MS analyses of the cuticular hydrocarbons (CHCs) of fungus-exposed versus unexposed bees. Our results demonstrated that exposed bees have altered cuticular hydrocarbons and are more easily accepted into foreign colonies than controls. Since CHCs are the main recognition cues in social insects, changes in their composition appear to affect nestmate recognition ability at the colony level. The acceptance of chemically unrecognizable fungus-exposed foragers could therefore favour forager drift and disease spread across colonies.

## Introduction

Bees are declining worldwide with considerable consequences on the pollination services they provide for crop production and the integrity of terrestrial ecosystems^1–3^. Agriculture intensification, pesticide exposure, and increased pressure of native and invasive parasites/pathogens have largely contributed to such decline^1,4–10^. As the adoption of intensive farming models aimed at feeding the world’s ever-growing population, cannot be separated from the massive use of agrochemicals^11,12^, in the last decades, an increasing effort has been dedicated to biological control strategies (biocontrol) to develop a more eco-friendly pest management approach in agriculture^13–15^. Nowadays, the use of microbial pathogens, parasites/parasitoids or predators to cope with insect pests, has partially replaced conventional synthetic plant-protection products in many countries^12,15–17^.

Among microbial pathogens, entomopathogenic fungi are extensively used as natural biocides in organic agriculture^18–23^. The worldwide-distributed *Beauveria bassiana* is a natural biocide widely used since the ‘80s^19,21,22^. This fungus attacks insects percutaneously: hydrophobic spores adhere to the cuticular hydrocarbons (CHCs) layer, germinate and penetrate the insect cuticle, killing the host a few days after the infection^20,21^. The frequent use of *B. bassiana* as a biocontrol agent is justified by its proved efficacy on target pests and by the low cost compared to conventional chemical insecticides; notably, potential side effects on humans and other non-target organisms have so far been considered as negligible^21^.

Honeybees often forage on crops biologically controlled with *B. baussiana*^24,25^ therefore, spores of the fungus are likely to adhere to the insect body during the repeated foraging flights^21,24,25^ but such *B. baussiana* spore contamination does not seem to represent a threat for bee survival^25,26^. The apparent low sensitivity of insect pollinators, such as honeybees and bumblebees, to this entomopathogenic agent has even encouraged the possible use of bee foragers as vectors to disseminate fungal spores on crops against target insect pests^24,25,27^ or the direct spread of *B. bassiana* spores inside hives to control *Varroa* mite populations^28,29^.

In recent years a strong debate has developed about potential side effects of chemical biocides on non-target species, especially insect pollinators^30–33^. Sub-lethal doses of insecticides and pesticides have been reported to influence behavioural traits, such as flight capacity, orientation ability and memory in honeybees and bumblebees, which in turn affect foraging performance or the return of foragers to their colony^31–40^. In this framework, beside the importance of toxicological tests to evaluate the survival of target and not-target insects, it is also crucial to assess the potential effects of biocides on the behaviour of non-target species as well as on the complex interactions at the colony level of non-target social species.

Social insects have evolved a number of behavioural adaptations to restrict the diffusion of diseases inside the colony, which go under the name of social immunity^41–43^. Among these adaptive responses to pathogens, social insects recognize individuals from other colonies as well as sick colony members and avoid, exclude, isolate or even kill them^41^. Cuticular hydrocarbons (CHCs), which are the cues at the basis of the recognition processes in social insects^44^, are also responsible for the discrimination of alien and sick or parasitized individuals^45–50^. The activation of the immune system, following an infection, can alter insect CHC profiles^45–47,50^. Consequently, biocontrol agents, including entomopathogenic fungi, can interfere with the colony recognition system in a subtle way by altering the individual chemical signature, as recently demonstrated in *Myrmica scabrinodi* naturally infected by ectoparasitic fungus *Rickia wasmannii*^51,52^ and in *Lasius neglectus* pupae artificially infected with *Metarhizium brunneum*^50^. If *B. bassiana* infection would cause similar alterations in bees, the allegedly safe biocontrol agents might have considerable effects on colony integrity and survival by impairing its recognition systems, therefore favouring the entering and the spread of parasites and pathogens among colonies vectored by drifting foragers.

In the present study, we investigated the effect of *B. bassiana* on the epicuticular hydrocarbon profile of fungus-exposed foragers and if these individuals are differently treated by guards at the hive entrance compared to unexposed conspecific individuals.

## Results

### Behavioural assays at the hive entrance

**Figure 1:**
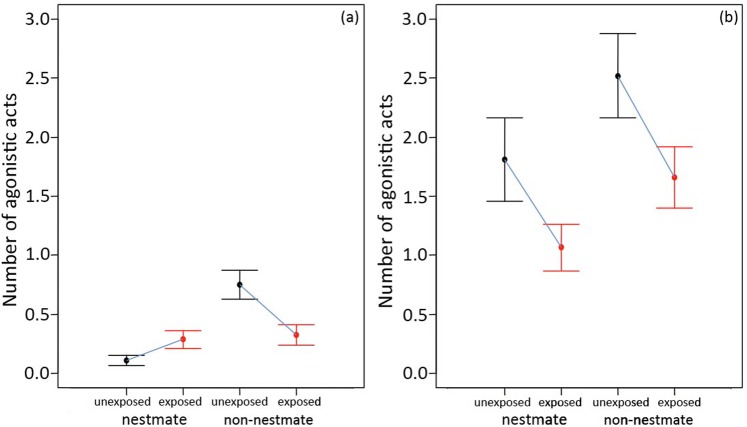
Mean number of agonistic acts (±standard error) received by unexposed nestmates, fungus-exposed nestmates, unexposed non-nestmates and fungus-exposed non-nestmates. (**a**) freeze-killed lures, (**b**) freely moving live bees.

Guard bees at the hive entrance responded differently to the presented stimuli in the bioassays with freeze-killed lures and live bees exposed to *B. bassiana* spores (see Methods, Fig. 1). As expected, nestmates were attacked less than non-nestmates but exposed bees were in general less attacked than non-exposed individuals (significant effects for colony membership and exposure in Table 1). An important difference also emerged between experiments with a lower reaction toward lures compared to live bees and with the occurrence of a significant interaction between colony membership and exposure in the case of lure experiment (see below). The different results in the two experiments are likely due to the fact that in our first experiment with lures we tested only guards’ response to the stimuli chemical components involved in recognition, while in the second bioassay, with live freely-moving bees, the behavioural interactions between guards and the presented stimuli were also maintained. However, although the overall aggressive response was lower in the bee-lure experiment (Fig. 1a), in both cases with freeze-killed lures and live bees, exposure to *B. bassiana* spores had a significant effect on recognition and a decrease in the guards’ aggressive response towards fungus-exposed non-nestmate foragers was observed.

**Table 1:** Results for a generalized mixed linear model applied to agonistic contacts (biting, stinging) received by honeybees (lures and live foragers) treated with a *Bauveria bassiana* suspension or a control solution (Exposure) presented on their own or to a different colony (Colony membership).

Variable	Estimate	Std. Error	z	P
**Freeze-killed lures**				
Colony membership	−1.938	0.617	−3.140	**0.002**
Exposure	−0.819	0.400	−2.050	**0.041**
Bees	−0.006	0.008	−0.800	0.425
Colony membership*Exposure	1.794	0.787	2.280	**0.023**
**Live foragers**				
Colony membership	−0.466	0.118	−3.960	**0.000**
Exposure	−0.265	0.109	−2.430	**0.015**
Bees	0.010	0.003	3.810	**0.000**
Colony membership*Exposure	−0.006	0.180	−0.030	0.975

#### Presentation of lures experiment

A mixed model GLM showed that guard bees had a lower aggressive reaction toward nestmates (Table 1) and that the fungus exposure lowered the aggression (Table 1). Furthermore, fungus exposure interacted with nestmate recognition and fungus-exposed bees (both nestmates and non-nestmates) were subjected to a similar and intermediate level of aggression between unexposed nestmates and non-nestmates (Table 1, Fig. 1a).

#### Live bees experiment

A mixed GLM revealed that also in this experiment, guard bees had a lower aggressive reaction toward nestmates (Table 1, Fig. 1b) and the fungus exposure lowered the aggression (Table 1). However, in the case of live bee exposure and colony membership (nestmate, non-nestmate) did not show a significant interaction and fungus-exposed nestmates received a much lower aggression than fungus-exposed non-nestmates (Table 1, Fig. 1b).

### Chemical analysis

To test whether bees showed any change in their epicuticular CHC profile after exposure to *Beauveria bassiana*, the CHC profile of fungus-exposed foragers was analysed with GC-MS. Analyses of the chromatograms for all the bees allowed the identification of 49 peaks corresponding to different compounds. The data set was reduced (see Methods) by cutting off 11 hydrocarbons; statistical analyses were thus performed on 38 compounds (Table 2). The identified compounds belong to four different classes: alkanes (N = 12), alkenes (N = 16), methyl-branched hydrocarbons (N = 1), esters between fatty acids and fatty alcohol (N = 8), plus one unidentified compound (Table 2).

**Table 2:** List of compounds analyzed in foragers either exposed to the entomopathogenic fungus *Beauveria bassiana* or unexposed, their relative average percentage (Mean) and standard error (SE) in the cuticular mixture.

Compound	Foragers		t	p
	Mean ± SE			
	Fungus-exposed N = 44	unexposed N = 41		
*n*-C_21_	8.96 ± 1.86	11.49 ± 2.26	−2.890	0.004*
*n*-C_22_	5.86 ± 1.63	7.39 ± 1.71	−2.626	0.010*
*n*-C_23_	603.34 ± 17.66	703.28 ± 16.31	−1.596	0.114
9-C_23:1_	77.72 ± 5.24	110.79 ± 6.52	−4.312	<0.0001*
7-C_23:1_	2.89 ± 1.40	3.59 ± 1.41	−1.651	0.102
C_23:2_	1.96 ± 1.35	5.01 ± 1.86	−5.689	<0.0001*
*n*-C_24_	21.91 ± 3.50	24.13 ± 3.03	−0.957	0.341
C_24:1_	3.61 ± 1.32	5.54 ± 1.40	−4.818	<0.0001*
2-meC_24_	1.14 ± 1.66	1.25 ± 1.21	−0.237	0.812
*n*-C_25_	726.53 ± 20.02	961.91 ± 39.33	−0.975	0.332
9-C^25:1^	159.46 ± 7.50	197.13 ± 7.99	−2.957	0.004*
7-C_25:1_	5.04 ± 1.77	11.69 ± 6.47	−1.047	0.297
C_25:2_	15.91 ± 2.57	24.07 ± 3.23	−4.450	<0.0001*
*n*-C_26_	16.47 ± 2.80	15.74 ± 2.43	0.497	0.620
Unidentified	5.84 ± 1.64	9.32 ± 2.26	−4.308	<0.0001*
*n*-C_27_	440.52 ± 16.84	345.26 ± 11.77	2.000	0.048**
C_27:1_a	45.13 ± 4.46	44.13 ± 4.35	0.252	0.801
C_27:1_b	14.17 ± 2.99	13.56 ± 2.72	0.339	0.735
C_27:2_a	3.30 ± 2.49	6.81 ± 3.62	−1.737	0.086
C_27:2_b	6.57 ± 2.01	10.14 ± 2.15	−3.793	0.0003*
*n*-C_28_	9.05 ± 2.53	8.73 ± 2.19	0.262	0.793
*n*-C_29_	242.36 ± 13.48	210.83 ± 10.98	0.934	0.352
C_29:1_	20.34 ± 4.37	20.94 ± 4.40	−0142	0.886
*n*-C_30_	5.71 ± 2.31	5.34 ± 2.49	0.298	0.765
*n*-C_31_	164.83 ± 12.04	156.45 ± 10.91	0.289	0.772
C_31:1_	39.67 ± 5.17	49.79 ± 6.71	−1.342	0.183
C_31:2_	53.52 ± 6.66	40.73 ± 4.42	1.708	0.091
*n*-C_33_	44.62 ± 9.38	27.37 ± 4.96	1.209	0.230
C_33:1_a	153.37 ± 11.13	127.73 ± 7.31	1.222	0.225
C_33:1_b	6.33 ± 3.04	3.75 ± 2.23	1.598	0.113
Oleic acid ester 1	27.83 ± 5.23	22.13 ± 4.16	1.147	0.254
Oleic acid ester 2	22.30 ± 4.98	28.09 ± 4.12	−1.244	0.216
Oleic acid ester 3	62.39 ± 8.10	69.65 ± 6.54	−0.598	0.551
Oleic acid ester 4	26.13 ± 6.29	23.06 ± 4.57	0.441	0.660
Palmitic acid ester 1	145.07 ± 11.41	89.75 ± 8.04	2.452	0.016**
Oleic acid ester 5	24.45 ± 4.89	41.44 ± 9.64	−1.169	0.245
Palmitic acid ester 2	95.09 ± 8.17	69.30 ± 7.41	1.951	0.054
Oleic acid ester 6	49.11 ± 6.13	53.96 ± 4.83	−0.707	0.481
Total amount	3358.08 ± 1221.17	3561.47 ± 1882.02	−0.595	0.553

Notes: In bold the compounds that were statistically different in the two treatments.

*Compounds significantly less abundant in the cuticle of fungus-exposed bees.

**Compounds significantly less abundant in the cuticle of unexposed bees.

No differences were found in the total amount of CHCs between unexposed and fungus-exposed foragers (GLM, t = −0.595, p = 0.5534). However, by considering the single compounds, we found that 6 alkenes (9-C_23:1_: t = −4.312, p < 0.0001; C_23:2_: t = −5.689, p < 0.0001; C_24:1_: t = −4.818, p < 0.0001; 9-C_25:1_: t = −2.957, p = 0.004; C_25:2_: t = −4.450, < 0.0001, p; and C_27:2_: t = −3.793, p = 0.0003) and 2 alkanes (n-C_21_: t = −2.890, p = 0.004; n-C_22_: t = −2.626, p = 0.010) were significantly lower in the fungus-exposed foragers than in the unexposed ones, while one alkane and one ester of palmitic acid was more abundant in the fungus-exposed bees (n-C_27_: t = 2.000, p = 0.048, palmitic acid ester 1: t = 2.452, p = 0.016) (Table 2).

**Figure 2:**
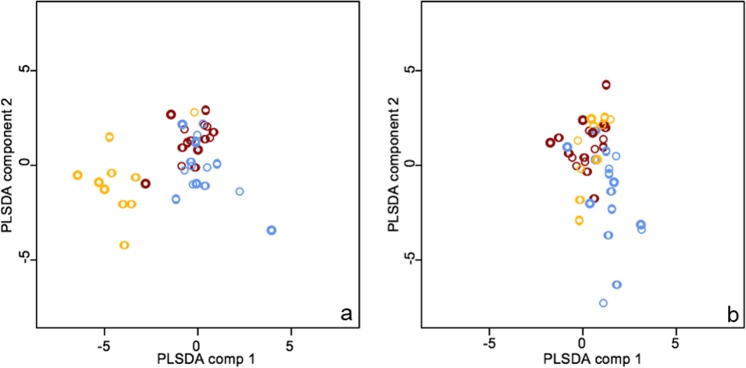
Scatterplot for the first two components of a Partial Least Squares Discriminant Analysis showing a good separation among unexposed bees (**a**) and a much weaker separation for exposed bees (**b**).

When plotted separately, the scores obtained by individuals of different colonies in a Partial Least Squares discriminant analyses, showed that unexposed individuals were characterized by a good separation based on the composition of their epicuticular lipids (Fig. 2a). Conversely, the scores obtained by fungus-exposed individuals revealed a much weaker diversification (Fig. 2b). Accordingly, a jackknife attribution of the specimens resulted in 63.4% of unexposed individuals correctly assigned to their colony against a 27.2% scored by fungus-exposed individuals. It must be noted that a random attribution of specimens to three colonies is expected to return a 33.3% of individuals correctly assigned by chance.

## Discussion

Our study demonstrated that the entomopathogenic fungus *Beauveria bassiana*, used as natural biocide in pest crop control, affects nestmate recognition in honeybees. As expected, guard bees at the hive entrance discriminated nestmate from non-nestmate foragers, being less aggressive toward unexposed nestmates. Interestingly, a reduction of the aggressive response was recorded when guards faced non-nestmate foragers experimentally exposed to the fungus *B. bassiana*. The decreased aggressive response towards exposed non-nestmates was observed when testing guards both with freeze-killed forager lures and with live foragers. With nestmate bees, instead, the guards’ reaction towards exposed individuals differed between the two experiments. In the experiment with freeze-killed lures, although the aggressive response was much lower than with live bees, exposed nestmates received a higher number of agonistic contacts when compared with unexposed ones at a rate that was comparable with exposed non-nestmates, Conversely, exposed live nestmates presented at the hive entrance received the lowest aggressive reactions by guards. Such a discordant result is most likely due to the different experimental protocols. In facts, it is plausible that in our first experiment with freeze-killed lures we tested exclusively the chemical cues involved in recognition. Guard bees might perceive the alteration in the chemical profile of their nestmates following fungus exposure which could prompt a relatively higher reaction towards them. In the case of live bees, fungus exposed individuals both nestmates and non-nestmates were less attacked compared to their unexposed controls and exposed nestmates received the lowest number of agonistic acts, while exposed non-nestmates were attacked at a rate that was comparable with unexposed nestmates. The lowered aggressive reaction of guards toward exposed bees regardless of colony membership is most likely due to the fact that live foragers presented at the hive entrance behaviourally interacted with the guards. If exposure to the fungus also altered their behaviour alongside their chemical profile, for example making exposed bees less reactive or aggressive, they could more easily sneak inside the colony. In facts, previous studies^53,54^ demonstrated that context or type of stimulus can have major effects on recognition. In the case of bees, guard are significantly more likely to make acceptance errors when tested in an unnatural context^53^, while the level of aggressive response can vary depending on the characteristics of presented stimulus as for example with *Polistes* wasps which show a lower degree of aggression when presented with only the cuticular fraction of a conspecific compared to a whole body wasp lure^54^. Overall, exposure to the fungus alters the CHCs of foragers bearing the mycosis after exposure to *B. bassiana* spores and this appears to be correlated to a significant change in the acceptance behaviour by guards towards fungus-exposed bees, especially increasing acceptance of exposed non-nestmates regardless of the experimental context, and such alteration.

Previous work on the CHCs dynamics on the tegument of insect hosts (*Ostrinia nubilalis* and *Melolontha melolontha* larvae) exposed to *B. bassiana* showed a high reduction (variable from 50% to 80% depending on the host) of the amount of such compounds within 96 h from infection^55^. Entomopathogenic fungi catabolize the long-chain hydrocarbons and penetrate inside the waxy cuticle of their insect hosts^56,57^ and such metabolic interactions may be responsible for the CHC alterations of mycosis-affected insects^50,56,57^.

By analysing the chemical profiles of fungus-exposed and unexposed foragers we found that differences between groups are mainly due to the different amount of six alkenes and two alkanes, which were consistently less abundant in fungus-exposed individuals, and one alkane and one ester of palmitic acid that was more abundant in the fungus-exposed bees rather than in the unexposed bees. The differential decrease in the alkene fraction in exposed individuals resulted in a homogenization of chemical profiles among colonies (Fig. 2) and it is likely at the basis of the observed reduction in aggressivity against exposed non-nestmates. This alteration of the hosts’ chemical profile may arise during the first phases of the infection process from the breakdown of hydrocarbons by *B. bassiana*^58^ or after infection due to an immune response affecting the synthesis of specific hydrocarbons^45,46^.

The selective reduction of specific alkenes is of particular interest, since it has been demonstrated that bees supplemented either with a synthetic mixture of alkenes or with the colony-specific alkenes fraction, are treated more aggressively by nestmate guards than control bees or nestmates applied either with a synthetic mixture of alkanes or with the colony-specific alkanes fraction^59^. Such findings indicate that alkenes play a crucial role in nestmate recognition in honeybees and that the relative increase of such compounds on the cuticle of incoming foragers elicits aggressive behaviour in guards, preventing them from recognizing nestmates^59^. Indeed, alkenes appear to be responsible for differences in the average chemical profile of different honeybee colonies as well as other social hymenoptera^60^. Moreover, alkenes have been found to be more easily learned and discriminated in PER (proboscis extension reflex) trained bees with respect to alkanes^60,61^. Thus, the lower amount of the six specific alkenes found on the cuticular profile of *B. bassiana*-exposed bees, with respect to unexposed ones, are most likely responsible for the higher degree of acceptance of guard bees towards fungus-exposed non-nestmate foragers with respect to unexposed non-nestmate ones.

Recent work has demonstrated that the selective increase of specific alkenes in pupae of the ant *Lasius neglectus* exposed to the enthomopathogenic fungus *Metarhizium brunneum* induce the killing of infected brood during the non-transmissible incubation period of the pathogen^50^. Our bees exposed to *Beauveria* showing a selective reduction in alkenes might instead be depleted of those compounds relevant for colony discrimination^60,61^. Such a modification interferes with the nestmate recognition system and guard bees at the hive entrance are unable to correctly assess foragers affected by the mycosis.

Several bee parasites have evolved adaptive strategies based on chemical mimicry to their host: *Varroa* mites, for instance, show an almost perfect colony specific mimicry with their host to avoid detection^62–65^. Chemical mimicry, replicating the colony specific pattern of CHCs, has also been reported for the death’s head hawkmoth *Acherontia atropos* and the parasitic fly *Braula coeca*^66,67^, and appears to be widespread in social insect parasites mimicking the host chemical profile to favour their spread and survival inside colonies^67^. Since *B. bassiana* is an unspecific insect pathogen, the modification of cuticular hydrocarbon profile observed in our fungus-exposed bees may be a mere by-product of the pathogen’s metabolism^56–58^ rather than an adaptive strategy evolved to infect honeybee colonies; nevertheless the selective decrease of cuticular olefins in our fungus-exposed bees could represent a serious threat for colony integrity.

Artificial infection with the non-specific pathogen *E. coli* also alters the cuticular profile of bee workers, but such changes did not consist of a selective reduction of the alkene fraction and the resulting alterations in cuticular profiles elicited an increase in the nestmates’ aggressive response towards bacteria-injected individuals^46^. A significant change in the CHC profile of honeybees has also been reported for workers infected with deformed wing virus (DWV) which are detected and removed from the colony^47^, however, also in this case, the chemical alteration involves different fractions of the cuticular compounds rather than the specific alkene fraction important for nestmate recognition^59^.

Overall, our results showed a clear disrupting effect of *B. bassiana* on the nestmate recognition system of honey bees, potentially favouring the drift of foragers to alien colonies and in turn the potential spread of parasites and pathogens. Indeed, an efficient nestmate recognition system is crucial for honey bee colonies as it limits drifting and robbing behaviour^68^, which are the main causes for the spread of infectious diseases, such as varroatosis, foulbrood and nosemosis^69–71^. Foragers carrying the fungal pathogen *Nosema ceranae* are even more prone to drift toward nearby hives than uninfected ones^71^. Given the use of *B. bassiana* as a natural biocide in organic agriculture and the possible use of pollinators as vectors for microorganisms in biocontrol^19,21,24,25^, future research should aim at investigating whether foragers exposed to the fungus spores show an altered behaviour (i.e. higher degree of drifting) with respect to unexposed bees.

Many recent efforts to limit the use of chemical pesticides and to adopt more sustainable agricultural models have led to a considerable increase in the use of bio-insecticides for pest management^13,14,17^. Although the adoption of natural biocides is desirable, we showed that treatments with the allegedly safe *B. bassiana* interfere with nestmate recognition in honeybees, possibly fostering inter-colony transmission of diseases. We thus warn that natural biocides used for pest management should not only be tested for their direct toxicity at the individual level on target and non-target species. The same attention should be dedicated to carefully testing for side effects on non-target ecologically relevant social insect species to understand the potential effects on those behavioural features linked to complex social organization determining the colony health status and survival.

## Methods

### Bee sample collection and experimental hives

Bee sample collections and behavioural field assays were carried out on hives of 3 experimental apiaries separated by at least 35 km from each other in the surroundings of Florence (Italy) (apiary A: 50 colonies; apiary B: 5 colonies; apiary C: 10 colonies). All the colonies used for the experiments were queen-right and housed in Dadant-Blatt boxes. All behavioural experiments and collections were carried out during spring and summer 2015 and 2016, between May and July.

### Isolation and culturing of *Beauveria bassiana* strains

*B. bassiana* (Balsamo) Vuillemin infective conidiospores (conidia) were isolated from the commercial product *Naturalis*® (Intrachem Bio Italia), a bioinsecticide based on living conidiospores of the naturally occurring *B. bassiana* strain ATCC 74040, isolated from *Anthonomus grandis* (Boheman), the cotton boll weevil. The formulated product contains at least 2.3 × 10^7^ viable spores/ml. Petri dishes with Malt Extract Agar (MEA, Oxoid) were plated with 100 μl of commercial product and incubated for three days at 30 °C. Fungal conidia emerging on plates were collected and re-suspended in a 0.01% Triton X-100-water solution at a concentration of 10^9^ spores/ml. Spore concentration was checked through vital count after plating different dilutions on MEA incubated for three days at 30 °C.

### Behavioural assays

Behavioural assays were performed, by using two different procedures, to test whether guard bees at the hive entrance are able to recognize fungus-exposed nestmate/non-nestmate foragers. The first bioassay consisted of presentation of lures at the hive entrance, where the importance of the mere chemical cues for recognition was tested. The second bioassay consisted of a more natural condition in which guard bees had to recognize fungus-exposed or unexposed live foragers induced to approach the hive entrance^72^; in this experiment, the behavioural components are maintained alongside the chemical ones. All the experiments were carried out in accordance with Italian laws and regulations.

#### Presentation of lures experiment

i) Lure preparation: forager bees were collected at the hive entrance while departing for their foraging flights (20 foragers per hive; 28 hives from apiary A; four hives from apiary C). Foragers were then brought to the laboratory, about half of the collected bees for each colony were exposed to the fungus by applying 1 μl of a 0.01% Triton X-100-water solution, containing about 10^6^ conidia of *B. bassiana*, on their thorax by using a 2 μl micropipette. We chose to use this concentration, corresponding to 10^9^ conidia/ml, since it appears similar or lower to those of the solution commonly sprayed in the field for pest biocontrol^19^ or pollinator biocontrol vector technology^24,25,27^. The other half of the foragers was applied with 1 μl of triton-water solution, containing no fungus conidia using the same procedure. Experimental and control bees were separated based on treatment and colony of origin in 7 cm Petri dishes, supplied with honey and water *ad libitum* for three days. A period of three days is sufficient for the fungus to reach the hemolymph and trigger an immune response^21,73^, potentially inducing detectable changes in epicuticular CHCs, which could in turn affect the bee guards’ response^46–49,72,74,75^. Foragers were kept in the dark, under controlled laboratory conditions (28–29 °C T, 55–65% RH); bees that died during treatment were daily removed from each dish. After three days, bees were killed by freezing and brought to the apiary A for field bioassays.
ii) Lure presentation: to perform the behavioural assay we followed a procedure similar to that used by Cappa *et al*.^76^: each of the selected colonies (N = 28) in apiary A was presented with four stimuli in a random sequence: two nestmate and two non-nestmate foragers from apiary C either unexposed or exposed to the fungus three days before the behavioural assays. Each lure was fixed to a small clip at the top of a 40 cm thin metal rod, slowly brought near to the hive entrance, and gently moved alongside the flight board contacting as many bees as possible in order to record their reactions^76^. Each presentation lasted 1 min starting from the first interaction of a bee with the lure. To avoid habituation effects, assays were performed on a same colony with at least a 15-min interval between each other. All the assays were carried out on a single day, between 10:00 and 16:00. All the assays were videotaped. Each lure was used only once and we carried out a total of 112 assays.

Videotapes were blind-watched in slow motion (0.25 s) by a viewer who noted the total number of bees interacting with each lure and the total number of agonistic contacts (biting, stinging) of resident bees towards the lure. As the lure was actively moved on the flight board to record the reactions of the highest possible number of bees, which could vary during the 1-minute assay, the average number of bees at the entrance was calculated by counting the number of bees on the flight board at the beginning and at the end of presentation^76^.

We used the “glmmadmb” function in the “glmmADMB” package (http://glmmadmb.r-forge.r-project.org/) to fit a Generalized Linear Mixed Model using a Poisson distribution. As predictor variables we used i) average number of bees at the entrance, ii) colony membership (alien *vs* nestmate), iii) exposure to *B. bassiana* as fixed factors together with the iv) interaction between colony merandom factor.

#### Live bee experiment

The following year, a different behavioural assay was performed by presenting live foragers to the colonies. These presented foragers were: a) unexposed nestmates, b) unexposed non-nestmates, c) fungus-exposed nestmates, d) fungus-exposed non-nestmates. Nestmate foragers (apiary B) and alien foragers (apiary C) were collected and treated following the same procedure described in the lure presentation experiment. Three days after the exposure with *B. bassiana*, both exposed and unexposed bees were individually placed into eppendorf tubes and carried to apiary A for the bioassay. Tubes were kept for several minutes in a ice-cooled styrofoam box before presentations, in order to calm down the experimental bees, therefore preventing them from fidgeting or flying away once freed from the tubes^57,72,74^. When the assay started, each focal bee was gently released on the landing board and was free to move, to interact with other bees, to enter the hive or fly away. Bee guards’ reactions were recorded for 3 minutes after the first interaction. When the focal bee entered the hive, or flew away without interacting with guards the assay was discarded. The videotaped assays were blind-watched to record the number and the duration of each agonistic interaction (biting, stinging) among focal bees and guards on the landing board. A total of 259 assays (108, 65, 86 respectively on hives a, b and c in apiary B) were performed (Table S1). We modelled the number of agonistic acts with the same design of the same Generalized Linear Mixed Model and contrast analysis as described above.

### Chemical analyses

#### Data collection

We collected 100 foragers from the three tested colonies a, b and c (at least 30 bees per colony) in the experimental apiary B. Following exactly the same procedure used for bioassays, we exposed half of these bees to *B. bassiana* conidia while the remaining half were used as control and received only the 0.01% Triton X-100-water solution. Experimental and control bees were separated by treatment and colony in 7 cm Petri dishes, supplied with honey and water *ad libitum* for three days. All the bee groups were maintained in the dark, under controlled laboratory conditions. Three days after the bees were killed by freezing for subsequent chemical analyses. Before the analyses, each bee was thawed, then the apolar fraction of the cuticular compounds of each worker (N = 85, 44 fungus-exposed, 41 unexposed controls) was extracted by washing the entire body for 10 min in 1 ml of pentane. The extracts were dried under a stream of nitrogen and then re-suspended in 10 µl of heptane with 70 ng/μl of heptadecane (*n-*C_17_) as internal standard. One μl of extract was injected in a Hewlett Packard (Palo Alto,CA, U.S.A.) 5890 A gas chromatograph (GC) coupled to an HP 5970 mass selective detector (using 70 eV electronic ionization source). A fused ZB-WAX-PLUS (Zebron) silica capillary column (60 m x 0.25 mm × 0.25 mm) was installed in the GC. The injector port and transfer line temperatures were set at 200 °C and the carrier gas was helium (at 20 PSI head pressure). The temperature protocol was: from 50 °C to 320 °C at a rate of 10 °C/min and the final temperature was kept for 5 minutes. Injections were performed in splitless mode (1 min purge valve off). Data acquisition and analysis were done using the Chem Station G1701 BA (version B.01.00) - Copyright © Hewlett-Packard 1989–1998.

CHCs were identified on the basis of their mass spectra, and equivalent chain length. For the preparation of the dataset used in statistical analysis we only included compounds quantified in at least 25% of specimens with the exception of compounds only identified in one of the treatment groups (exposed/unexposed). We used Mixed-effects models for repeated-measures ANOVA (glmmPQL function of the MASS R package) to evaluate differences both in the total amount of cuticular hydrocarbons present in the extracts and in the amount of single compounds between fungus-exposed and control bees; colony membership was included as a random factor. Multivariate analyses have been applied to the dataset to verify the possibility of attributing fungus-exposed and unexposed specimens to their colony based on chemical composition. With this aim we first performed a Partial Least Square Discriminant analysis using colony membership as a priori grouping variable. This allowed us to visualize the general pattern of similarity among individuals. The possibility to attribute specimens to their colonies has been tested by a jackknife procedure where a Partial Least Square Discriminant analysis has been performed on all the uninfected specimens but one. Then the colony membership of the excluded specimen and of one fungus-exposed individual (in random order) has been predicted on the basis of their CHCs composition. We used the percentage of correctly attributed cases as a measure of the possibility to blindly attribute fungus-exposed and unexposed individuals to their colonies.

## Supplementary information

Table S1

Supplementary Dataset 4

Supplementary Dataset 5

Supplementary Dataset 3

Supplementary Dataset 1

Supplementary Dataset 2

## Data Availability

Data files and R script used are uploaded alongside supplementary material.
