# Supplementary material for: Natural biocide disrupts nestmate recognition in honeybees
Source: Sci Rep. 2019 Feb 28;9:3171. doi: 10.1038/s41598-019-38963-3 (PMC6395671; doi:10.1038/s41598-019-38963-3)
Supplement: Supplementary file 1 — Table S1 [file 41598_2019_38963_MOESM1_ESM.docx]

**Natural biocide disrupts nestmate recognition in honeybees**

F. Cappa^1^, I. Petrocelli^1^, F. R. Dani^1^, L. Dapporto^1^, M. Giovannini^1^, J. Silva-Castellari^1^, S. Turillazzi^1^ & R. Cervo^1^

1. Università degli studi di Firenze, Dipartimento di Biologia, Via Madonna del Piano 6, 50125, Sesto Fiorentino, Firenze

Table S1. Number of assays performed for each focal hive (a, b, c).

|  | Fungus-exposed Nestmate | Fungus-exposed Non-nestmate | Unexposed Nestmate | Unexposed Non-nestmate | TOT |
| --- | --- | --- | --- | --- | --- |
| a | 22 | 32 | 27 | 27 | 108 |
| b | 15 | 16 | 16 | 18 | 65 |
| c | 20 | 26 | 20 | 20 | 86 |
